# Pathogens vectored by the tick, *Dermacentor reticulatus*, in endemic regions and zones of expansion in Poland

**DOI:** 10.1186/s13071-015-1099-4

**Published:** 2015-09-24

**Authors:** Ewa J. Mierzejewska, Agnieszka Pawełczyk, Marek Radkowski, Renata Welc-Falęciak, Anna Bajer

**Affiliations:** Department of Parasitology, Institute of Zoology, Faculty of Biology, University of Warsaw, 1 Miecznikowa Street, 02-096 Warsaw, Poland; Department of Immunopathology of Infectious and Parasitic Diseases, Medical University of Warsaw, 3c Pawińskiego Street, 02-106 Warsaw, Poland

**Keywords:** *Dermacentor reticulatus*, Pathogens, *Babesia canis*, *Rickettsia raoulti*, *Borrelia burgdorferi* s.l, Poland, expansion

## Abstract

**Background:**

*Dermacentor reticulatus* plays an important role in the maintenance of pathogens of medical and veterinary importance in the environment. Currently two isolated populations of *D. reticulatus* are present in Poland –Western and Eastern. The range of the Eastern population covers endemic areas in eastern Poland but this population is expanding westwards creating an expansion zone in the centre of the country. The expansion zone in western Poland is occupied by the recently discovered Western population, spreading eastwards.

**Methods:**

Questing adult ticks (*n* = 2585) were collected in 2012–2014 in endemic regions of north-eastern (Warmińsko-Mazurskie Voivodeship) and central Poland (Masovian Voivodeship) and in the expansion zones in central and western Poland, in the region between the Vistula River and the western border of the country. Amplification of *Babesia*, *Rickettsia* spp. and *Borrelia burgdorferi* sensu lato DNAs was performed using specific starters. RNA of the TBE virus was detected using RT-PCR and representative PCR products were sequenced and compared with sequences deposited in GenBank.

**Results:**

Of the total 2585 examined ticks, 1197 (46.3 %) were infected with at least one pathogen. Overall prevalence of pathogens was 4.18 % (108/2585) for *Babesia* spp.*,* 44.10 % (1140/2585) for *Rickettsia* spp., 0.09 % (1/1107) for *Borrelia afzelii* and 7.6 % (7/92) for TBEV. Sequence analysis of DNA showed 99.86 % similarity to *R. raoulti* and 99.81 % to *B. canis.* One male from north-eastern Poland was infected with *B. microti.*

Prevalence of *R. raoulti* was highest in the Western population (52.03 %) and lowest in the Eastern population in north-eastern Poland (34.18 %). *Babesia canis* was not detected in 592 ticks collected in the Western population, while in the Eastern population overall prevalence was 5.42 %. There were significant differences in the prevalence of *B. canis* between tick samples from northern (0.68 %), central (1.18 %) and southern (14.8 %) areas of the expansion zone in central Poland.

**Conclusions:**

Our study found significant differences between the range and prevalence of vectored pathogens in *D. reticulatus* from the endemic areas and newly inhabited expansion zones. The differences were likely associated with the different time of settlement or ‘source’ of ticks populations, the Eastern and the Western one.

## Background

*Dermacentor reticulatus* (Fabricius, 1974) is the main vector of *Babesia canis* [1–3], the aetiological agent of canine babesiosis, responsible for one of the most threatening infectious diseases of dogs in endemic regions [4–6]. *D. reticulatus* has been observed to spread in many European countries over the last two decades, and this is a subject of considerable concern for the veterinary services. In some countries considered to be free of *D. reticulatus*, autochthonous cases of canine babesiosis were the first sign of appearance of this tick, e.g., Belgium [7] and Netherland [8]. Until the 1990’s foci of this tick in Poland were known to exist almost exclusively in north-eastern parts of the country [9–12]. The area between the Vistula River and the western border of the country was considered to be a territory free of *D. reticulatus,* splitting its range into Western European and Eastern macro regions [13–15]. In the late 1990’s *D. reticulatus* ticks were found on dogs in the Masovian Voivodeship, east of the Vistula River (own unpublished data). Currently these areas are considered to be endemic for *D. reticulatus* and canine babesiosis [5, 6]. At the beginning of XXI^th^ century canine babesiosis had spread further and was commonly diagnosed in dogs living west of the Vistula River, in the vicinity of Warsaw [4, 5, 16]. Zygner and Wędrychowicz [17] confirmed the frequent occurrence of *D. reticulatus* in the Warsaw region, first on dogs and subsequently these ticks were collected from vegetation [18, 19]. Recently new foci of *D. reticulatus* have been found in western Poland, in the area historically free of this tick [20–22].

In our recent study, we discovered a large and stable population of this tick in western Poland [23]. Moreover, the monitoring of *D. reticulatus* in the extensive region between the Vistula River and the western border of the country in 2012–2014 has confirmed expansion of this species westwards, west of the Vistula River and eastwards in western Poland [23]. Those two expansion zones are separated by ‘the gap’ in the range – the area where *D. reticulatus* has so far never been found. This gap splits populations of this species in Poland into two separated populations - Western and Eastern. The discoveries of a large population in western Poland and associated active expansion zones led to pertinent questions about the range and prevalence of important tick-borne pathogens (TBP’s) vectored by ticks from different regions of the country.

The host spectrum for *D. reticulatus* is wide and differs at every life stage. Larvae and nymphs are endophilic and parasitize small mammals living in burrows. Adults are exophilic and feed on large ungulates, carnivores, horses or wild boars [24–28]. This tick species attacks humans very rarely [29, 30]. However, *D. reticulatus* plays an important role in the maintenance of TBP's of veterinary and medical importance in the environment and their transmission between vertebrate hosts that are susceptible to infection or serve as efficient reservoirs. Based on detection of pathogen specific DNA, micro-organisms known to cause diseases of animals and humans, have been detected in ticks of this species, e.g., *Rickettsia* spp., TBEV, *Borrelia burgdorferi* s.l., *Anaplasma phagocytophlium, Bartonella* spp*. Coxiella burnetti*, *Francisella tularensis* [31–34]. Nevertheless, the prevalence of some of these pathogens can be very low and the status of *D. reticulatus* as their vector has been questioned [35–37]. In recent years a high prevalence of tick-borne encephalitis virus (TBEV) has been demonstrated in ticks in two studies from eastern and central Poland [38, 39], a virus to which dogs are known to be susceptible, as first demonstrated by Weissenböck et al. [40] and then confirmed by Bajer et al. [41].

As far as we are aware, the prevalence of pathogens of veterinary and medical importance in ticks has not been investigated in western Poland and studies on the infection rate of pathogens other than TBEV [39] in questing ticks from central Poland have not yet been conducted on a large scale. The discovery of several new locations of *D. reticulatus* in two expansion zones west of the Vistula River and in western Poland [23] has created a unique opportunity to study the prevalence of pathogens in two distinct, geographically separated populations of this tick species. Importantly, the first recorded occurrence of *D. reticulatus* in the eastern and western regions was markedly different, raising questions about the contrasting prevalence rates of pathogens in ticks from regions that have varied over the years in the duration over which they have been inhabited by this tick species.

The aims of the present study were (1) to compare prevalence of *Babesia* and *Rickettsia* spp. between two tick populations and between endemic and newly inhabited regions of Poland, (2) to assess prevalence of TBEV in endemic regions of central Poland (Masovian Voivodeship) and (3) to study the role of *D. reticulatus* in transmission of *B. burgdorferi* s.l.

## Methods

### Collection sites

**Fig. 1:**
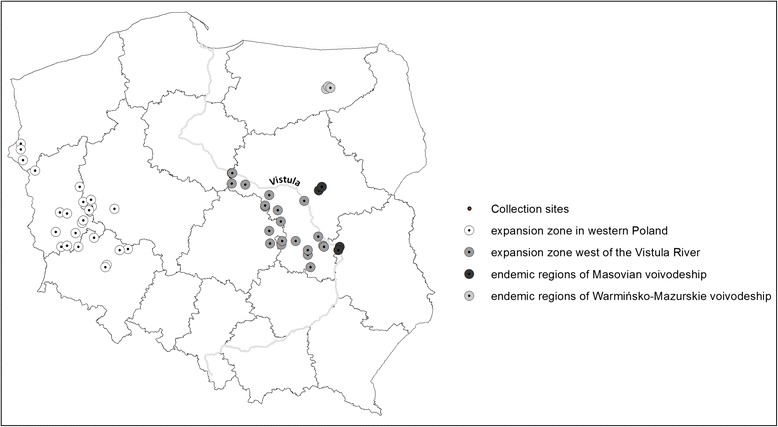
The location of all the collection sites utilized in the current study

Adult questing ticks were collected from September 2011 to May 2014. Additionally, 96 ticks (52 females and 44 males) collected in spring 2009 in Kury (Eastern population, endemic region, east of the Vistula River) were included in the study. Ticks were collected in typical habitats: fallow lands and meadows covered by vegetation higher than 60 cm, located close to water reservoirs and water courses. Sites for tick collection were selected across Poland in four regions that differ in time of appearance/ settlement of *D. reticulatus* ticks (Fig. 1). Two of these regions were part of the Eastern population, situated in endemic areas of the Warmińsko-Mazurskie Voivodeship where the earliest reported foci of *D. reticulatus* have been documented and Masovian Voivodeship, east of the Vistula River, where presence of this tick species is known only from the late 1990’s. A further two regions were situated in two expansion zones – territories west of the Vistula in central Poland (expanding part of the Eastern population known to exist only from the beginning of XXI^th^ century) and areas in western Poland (recently discovered eastwardly expanding Western population). All the sites utilized in this study have been described in detail in Mierzejewska et al. [23]. Collection sites (*n* = 39) in endemic regions (*n* = 9) and expansion zones (*n* = 30) are shown on Fig. 1. A detailed list of adult *D. reticulatus* ticks collected at each site is shown in Table 1.

**Table 1:** Number of adult ticks *D. reticulatus* collected at each site

Region	Site of ticks collection		Year, season and sex of tick												Total
			2011			2012			2013			2014			
			A		∑	S+A		∑	S+A		∑	S		∑	
			♀	♂		♀	♂		♀	♂		♀	♂		
Masovian endemic	Dąbrowica		24	16	40	10	16	26	42	27	69	40	24	64	199
	N 52.357819														
	E 21.453904														
	Kury		3	4	7	12	16	28	nc	nc	nc	17	10	27	62
	N 52.413677														
	E 21.512028														
	Miąse		nc	nc	nc	nc	nc	nc	10	5	15	0	1	1	16
	N 52.397716														
	E 21.445945														
	Stoski		4	10	14	49	56	105	140	41	181	40	11	51	351
	N 52.409226														
	E 21.509125														
	∑		31	30	61	71	88	159	192	73	265	97	46	143	628
Warmińsko-Mazurskie endemic	Dziubiele		12	8	20	28	31	59	6	10	16	32	39	71	166
	N 53.814619														
	E 21.704191														
	Łuknajno		17	30	47	27	5	32	12	11	23	nc	nc	nc	102
	N 53.802309														
	E 21.644787														
	Osa		18	16	34	7	0	7	19	15	34	22	6	28	103
	N 53.820064														
	E 21.650590														
	Stawek		8	14	22	5	3	8	12	5	17	nc	nc	nc	47
	N 53.794089														
	E 21.616050														
	Urwitałt		8	10	18	8	11	19	14	5	19	nc	nc	nc	56
	N 53.809755														
	E 21.645224														
	∑		63	78	141	75	50	125	63	46	109	54	45	99	474
Expansion zone in central Poland	Northern area	Dąb Polski	nc	nc	nc	nc	nc	nc	1	3	4	nc	nc	nc	4
		N 52.601013													
		E 19.387562													
		Koszelówka	nc	nc	nc	nc	nc	nc	10	9	19	30	24	54	73
		N 52.441638													
		E 19.713322													
		Plecewice	nc	nc	nc	nc	nc	nc	9	5	14	23	34	57	71
		N 52.284977													
		E 20.274725													
		∑	nc	nc	nc	nc	nc	nc	20	17	37	53	58	111	148
	Central area	Międzyborów	nc	nc	nc	23	15	38	7	4	11	27	21	48	97
		N 52.066697													
		E 20.466948													
		Piekary	nc	nc	nc	nc	nc	nc	nc	nc	nc	4	2	6	6
		N 51.892776													
		E 20.532884													
		Siekierki	nc	nc	nc	49	47	96	97	76	173	28	24	52	321
		N 52.205007													
		E 21.084998													
		∑	nc	nc	nc	72	62	134	104	80	184	59	47	106	424
	Southern area	Pieńki	nc	nc	nc	nc	nc	nc	0	0	0	4	3	7	7
		N 51.23602													
		E 21.244804													
		Kociołki	nc	nc	nc	nc	nc	nc	6	5	11	14	12	26	37
		N 51.542445													
		E 21.557737													
		Korzeń	nc	nc	nc	nc	nc	nc	7	6	13	14	0	14	27
		N 51.631894													
		E 20.896111													
		Nowe Miasto n/Pilicą	nc	nc	nc	nc	nc	nc	9	8	17	7	8	15	32
		N 51.629796													
		E 20.568063													
		Owadów	nc	nc	nc	nc	nc	nc	14	4	18	40	27	67	85
		N 51.488246													
		E 21.175258													
		Radom	nc	nc	nc	nc	nc	nc	4	5	9	7	0	7	16
		N 51.429317													
		E 21.165899													
		Rawa Maz.	nc	nc	nc	nc	nc	nc	nc	nc	nc	6	3	9	9
		N 51.763742													
		E 20.274813													
		Ryczywół	nc	nc	nc	nc	nc	nc	5	3	8	nc	nc	nc	8
		N 51.690792													
		E 21.417891													
		Rzeczyca	nc	nc	nc	nc	nc	nc	nc	nc	nc	2	0	2	2
		N 51.571705													
		E 20.237232													
		∑	nc	nc	nc	nc	nc	nc	45	31	76	94	53	147	223
Expansion zone in western Poland	Northern area	Chojna	nc	nc	nc	2	5	7	13	14	27	nc	nc	nc	34
		N 52.941048													
		E 14.443807													
		Mieszkowice	nc	nc	nc	nc	nc	nc	8	5	13	nc	nc	nc	13
		N 52.757022													
		E 14.504899													
		∑	nc	nc	nc	2	5	7	21	19	40	nc	nc	nc	47
	Central area	Konotop	nc	nc	nc	nc	nc	nc	1	0	1	nc	nc	nc	1
		N 51.925143													
		E 15.897403													
		Kościan	nc	nc	nc	nc	nc	nc	6	6	12	nc	nc	nc	12
		N 52.096831													
		E 16.635880													
		Krępa	nc	nc	nc	40	41	81	0	0	0	18	16	34	115
		N 52.010798													
		E 15.542329													
		Nietków	nc	nc	nc	1	0	1	nc	nc	nc	nc	nc	nc	1
		N 52.030810													
		E 15.345025													
		Obra	nc	nc	nc	0	0	0	9	3	12	4	0	4	16
		N 52.069111													
		E 16.041058													
		Polkowiczki	nc	nc	nc	26	16	42	nc	nc	nc	nc	nc	nc	42
		N 51.549555													
		E 15.512411													
		Popowice	nc	nc	nc	21	11	32	42	38	80	34	14	48	160
		N 51.755451													
		E 15.251825													
		Przemków	nc	nc	nc	48	27	75	nc	nc	nc	nc	nc	nc	75
		N 51.534175													
		E 15.778345													
		Rudawica	nc	nc	nc	3	0	3	nc	nc	nc	nc	nc	nc	3
		N 51.536233													
		E 15.396157													
		Sierczynek	nc	nc	nc	nc	nc	nc	3	4	7	nc	nc	nc	7
		N 52.380312													
		E 15.797783													
		∑	nc	nc	nc	139	95	234	61	51	112	56	30	86	432
	Southern area	Kawice	nc	nc	nc	nc	nc	nc	60	16	76	21	12	33	109
		N 51.244003													
		E 16.428092													
		Klucze	nc	nc	nc	2	0	2	nc	nc	nc	nc	nc	nc	2
		N 51.667118													
		E 16.149976													
		Lubiąż	nc	nc	nc	0	2	2	nc	nc	nc	nc	nc	nc	2
		N 51.260855													
		E 16.461059													
		∑	nc	nc	nc	2	2	4	60	16	76	21	12	33	113
Total			94	108	202	361	302	663	566	333	899	434	291	725	2489

nc – ticks have been not collected, A - autumn, S - spring

### Tick collection

Ticks were collected by conventional dragging in the morning (9:00–12:00) or in the afternoon (from 15:00 to the dusk) from the middle of March to early May and from the middle of September to the beginning of November each year (a detailed description is provided in Mierzejewska et al. 2015 [23]). Collected ticks were preserved in 70 % ethanol or were stored in a cooler at a temperature of +8 °C. Tick species and sex were determined using a stereo microscope.

### DNA and RNA isolation

DNA was extracted from 2585 ticks using the QIAamp DNA mini kit (QIAGEN, Germany) following the manufacturer’s instructions. Every tick was cut by half along its longitudinal axis so as to provide the optimal weight of each sample recommended by the manufacturer. Total DNA was eluted in 160.0 μL of elution buffer. Extracted DNA was stored in −20 °C for further procedures. RNA was extracted from 92 ticks collected in endemic regions of the Masovian Voivodeship, east of the Vistula River. RNA was extracted from live ticks using AllPrep DNA/RNA Mini Kit (QIAGEN). To obtain cDNA, 5.0 μL of the RNA preparation were reverse-transcribed in a 15.0 μL of final reaction volume containing 3.0 μl of 25 mM MgCl_2_ (Thermo Scientific), 1.5 μl of 10xPCR Buffer, 1.5 μl of 10 mM dNTP (Invitrogen, USA), 0.5 μl of 50.0 μM random hexamer primers (Roche, Indianapolis, USA), 0.75 μl of 0.1 M DTT (Invitrogen), 20.0 U M-MLV reverse transcriptase (Invitrogen). The reverse transcription was performed at 37 °C for 30 min, at 95 °C for 5 min and at 4 °C for 5 min. Both RNA and cDNA were stored in −80 °C for further analysis.

### DNA amplification

The extracted DNA was subjected to PCR with the specific primers. Primers and reaction conditions were as previously described by the original authors mentioned in detailed description below. Each reaction was carried out in a 20.0 μl of the final PCR mixture volume containing 0.33 mM dNTPs (Eurobio, Lille, France), 2.0 mM MgCl_2_, 1 × PCR buffer, 1.0 U DreamTaq polymerase (Fermentas). The amount of primers used and the volume of template DNA varied between protocols for different pathogens as described in detail below. All negative controls were performed in the absence of template DNA. Amplicons were visualized with Midori Green stain (Nippon Genetics Europe GmbH) following electrophoresis in 2 % agarose gels. Amplicons were purified and sequenced by a private company (Genomed S.A., Poland). The resulting sequences were compared with sequences deposited in the GenBank database (http://blast.ncbi.nlm.nih.gov/Blast.cgi). DNA sequence alignments were conducted using the program Bioedit 7.1.

### *Babesia* spp. and *Rickettsia* spp.

The DNA amplification was carried out in 20 μl of the final PCR mixture contained 1.0 μM of each primer. In the protocol for *Rickettsia* spp. primers CS409/ Rp1258 [42] were used to produce a ~750 bp fragment of *glt*A gene. Nested - PCR reaction targeting 18S rRNA was performed to detect genetic material of *Babesia* spp. In the first reaction with the outer primers CRYPTO R/ CRYPTO F [43] fragment of length ~1200 bp was amplified. For the second reaction two different pairs of primers were used to obtain maximum reliable results: Bab GR2/ Bab GF2 [44] or Piro A/ Piro B [45] to produce a ~550 bp or ~400 bp fragment respectively. For the protocol for *Rickettsia* spp. and for the first reaction with the outer primers in the protocol for *Babesia* spp., the template DNA volume was 2.0 μl. The second reaction with the inner primers in the protocol for *Babesia* spp. was carried out with 1.0 μl of the post-first reaction mixture as the template DNA. The positive control in the *Babesia* spp. protocol was the DNA of *Babesia microti* Kings Collage strain [46]. The positive control in the *Rickettsia* spp. protocol was the DNA of *R. helvetica* [47].

### *Borrelia burgdorferi* s.l.

Detection of *B. burgdorferi* s.l. was conducted in DNA extracted from 1107 ticks: 262 ticks collected in endemic regions in central Poland, 192 in endemic regions in Warmińsko-Mazurskie Voivodeship, 427 in non-endemic regions in central Poland (expansion zone west of the Vistula River), 228 in non-endemic regions in western Poland. For detection of this species, nested - PCR reaction targeting *fla* gene fragment (774 bp) was performed in 20.0 μl of the final PCR mixture contained 0.2 μM of each primer [48]. For the first reaction with outer primers 132f/ 905r the template DNA volume was 2.0 μl. In second reaction with inner primers 220f/ 824r, 1.0 μl of 10-times diluted post-first reaction mixture was used as the template DNA to produce the final product of 605 bp.

The positive control was the DNA of *B. burgdorferi* sensu stricto kindly provided by Dr. Nataliia Rudenko and Dr. Maryna Golovchenkofrom Biology Centre AS CR, Institute of Parasitology, Ceske Budejovice, Czech Republic.

### TBEV

For detection of TBEV, cDNAs were screened by Real-Time PCR. Reactions were carried out using the LightCycler FastStart DNA Master Sybr Green I Kit (Roche) in a total volume of 20.0 μl containing 5.0 μM of each primer TBEV_2F or TBEV_2R [49] and 2.0 μl of template cDNA. The final product was 195 bp fragment of 16S rRNA. The positive control was the RNA of TBEV Sofin strain kindly provided by Dr. Bernd Hoffmann from the Institute of Diagnostic Virology, Friedrich Loeffler Institute, Greifswald-Insel Riems, Germany.

### Statistical analysis

The prevalence of *B. canis* and *R. raoulti* were analyzed by maximum likelihood techniques based on log linear analysis of contingency tables, implemented by the software package, SPSS v. 21. Four statistical models were constructed to analyze the effect of different categories of regions on the prevalence of *B. canis* and *R. raoulti*: (1) between Eastern and Western tick populations (1, 2); (2) between endemic or non-endemic region (1, 2); (3) between regions inhabited by *D. reticulatus* in different time–new expansion zones in western or central Poland, old endemic regions in central or north-eastern Poland (1–4 categories); (4) between northern, central and the southern areas (1–3) among both expansion zones.

All factors were fitted into a full factorial model. Beginning with the most complex model, involving all possible main effects and interactions, those combinations not contributing significantly to explaining variation in the data were eliminated stepwise (backward selection procedure), beginning with the highest-level interaction [50]. A minimum sufficient model was then obtained, for which the likelihood ratio of *χ*^2^ was not significant, indicating that the model was sufficient in explaining the data.

### New nucleotide sequences

New nucleotide sequences have been deposited in GenBank with the accession numbers KT272401 for 18S rRNA of *B. canis* and KT277489 for *glt*A of *R. raoulti*.

### Ethical approval

The study was approved by the National Science Center (NCN).

## Results

### Prevalence of pathogens in questing D. reticulatus

Of the total 2585 examined ticks, 1197 (46.3 %) were infected with at least one pathogen. Overall, the prevalence of detected pathogens was 4.18 % (108/2585) for *Babesia* spp.*,* 44.10 % (1140/2585) for *Rickettsia* spp., 0.09 % (1/1107) for *Borrelia afzelii* and 7.6 % (7/92) for TBEV. The summary of results of all molecular analysis is provided in Table 2.

**Table 2:** Prevalence of *B. canis* and *R. raoulti* in *D. reticulatus* collected in areas covered by the Eastern and the Western population

Population	*B. canis*	*R. raoulti*	Endemicity	*B. canis*	*R. raoulti*	Time of inhabitation	*B. canis*	*R. raoulti*	Area of expansion zone	*B. canis*	*R. raoulti*
	positive/total (%)	positive/total (%)		positive/total (%)	positive/total (%)		positive/total (%)	positive/total (%)			
Eastern population	108/1993 (5.42)	832/1993 (41.75)	endemic regions	69/1198 (5.76)	451/1198 (37.65)	Warmińsko-Mazurskie	11/474 (2.32)	162/474 (34.18)		positive/total (%)	positive/total (%)
						Masovian	58/724 (8.01)	289/724 (39.92)			
			non-endemic regions (expansion zones)	39/1387 (2.81)	689/1387 (49.68)	west of the Vistula River	39/795 (4.91)	381/795 (47.92)	northern	1/148 (0.68)	37/148 (25.0)
									central	5/424 (1.18)	236/424 (55.66)
									southern	33/223 (14.80)	108/223 (48.43)
Western population	0/592 (0)	308/592 (52.03)				western Poland	0/592 (0)	308/592 (52.03)	northern	0/47 (0)	33/47 (70.21)
									central	0/432 (0)	212/432 (49.07)
									southern	0/113 (0)	63/113 (55.75)
Total							108/2585 (4.18)	1140/2585 (44.10)			

### Genotyping and diversity of pathogens

The 550 bp fragment of the 18S rRNA gene of *Babesia* spp. was detected in 108 isolates from ticks collected from the Eastern population. Fifty three PCR products of reaction with Bab GR2/GF2 primers and 5 with Piro A/Piro B primers were sequenced. Thirty two sequences were derived from ticks collected in the expansion zone in central Poland (west of the Vistula River), and 23 and 3 in the endemic regions of Masovian and Warmińsko-Mazurskie voivodeships, respectively. Forty sequences derived from the reaction with Bab GR2/GF2 primers were identical and showed 99.81 % (520/521) similarity to *B. canis* (GenBank: FJ209024, AY072926, EU622793, AY962187) derived from dogs in Croatia [51], Italy [52], Poland (*B. canis* isolate 2) [53] and from southwestern Siberia [54]. In a further 14 identical sequences, two unresolved positions were detected at position 610 (A↔G) and position 611 (A↔G) of the complete 18S rRNAgene (AY072926), indicating the presence of different alleles of *B. canis*. One isolate from a male tick collected in Łuknajno (endemic regions of Warmińsko-Mazurskie Voivodeship, north-eastern Poland) was identified as *B. microti* showing 99.81 % (512/513) similarity to *B. microti* clone Omsk-vole110 (GenBank: KC581934) derived from a bank vole in Western Siberia in Russia [55]. This is a genetic variant of the nonpathogenic *Babesia microti* Munich strain.

Of the 1140 amplicons of *Rickettsia* spp., 127 were sequenced and analyzed: 17 and 8 were obtained from ticks collected in endemic regions of Masovian and Warmińsko-Mazurskie voivodeships, respectively, 23 and 79 (4 sites) from the expansion zones of the Eastern and Western populations, respectively. All obtained sequences were identical and showed 99.86 % (717/718) similarity to *R. raoulti* Krasnoobsk strain (GenBank: KM288483).

Although bands of the correct size for *Borrelia fla* gene fragment (about 600 bp) were obtained for ten DNA isolates, only one PCR product was successfully sequenced. Therefore, only one female tick collected in Kury (endemic region, Masovian Voivodeship) was considered as positive. The sequence obtained showed 99.81 % (554/555) similarity to *B. afzelii* genotype (GenBank: DQ016619).

### Co-infections in questing ticks

In total, co-infections with two pathogens were identified in 59 (2.3 %) ticks. However, prevalence of co-infections exceeded 3 % (59/1993) among the Eastern tick population. Co-infections with three or more species of pathogens were not detected. The majority of co-infections (53 ticks) were with *R. raoulti* and *B. canis*. Double infections were more common in the endemic region of Masovian Voiodeship (29 ticks) than in the Warmińsko-Mazurskie (3 ticks). In the expansion zone west of the Vistula River, 21 ticks were co-infected with *B. canis* and *R. raoulti*. Additionally, six ticks from the endemic regions of the Masovian Voivodeship were infected with *R. raoulti* and TBEV.

### *Comparison of* B. canis *infection in questing ticks from different regions*

*Babesia canis* DNA was found only in ticks from the Eastern *D. reticulatus* population (108/1993 = 5.42 %). All 592 isolates from the Western tick population were negative (*χ*^2^ = 35.38, *df =* 1, *p <* 0.001). The differences in prevalence of *B. canis* between regions among the Eastern population inhabited in different years were also significant (*χ*^2^ = 71.51, *df* = 3, *p* < 0.001). The highest prevalence was found in ticks from the endemic regions of the Masovian Voivodeship in central Poland (58/724 = 8.01 %) and the lowest in the endemic regions of Warmińsko-Mazurskie Voivodeship in north-eastern Poland (11/474 = 2.32 %). In the expansion zone west of the Vistula River 4.91 % (39/795) of ticks were infected.

Interestingly, in the expansion zone west of the Vistula River, the prevalence of *Babesia* spp. changed significantly in a north-to-southerly direction (*χ*^*2*^ = 65,71, *df* = 2, *p <* 0.001). In northern and central areas the prevalence of *Babesia* spp. was relatively low- 0.68 % (1/148) and 1.18 % (5/424), respectively. In the southern region the number of positive ticks was unexpectedly high (33/233), resulting in the highest prevalence recorded (14.8 %).

### *Comparison of* Rickettsia spp. *infection in questing ticks from different regions*

In contrast to *B. canis*, the prevalence of *R. raoulti* infection was similar in the Western and the Eastern *D. reticulatus* populations: 52.03 % (308/592) and 41.75 % (832/1993), respectively (NS).

Prevalence of *R. raoulti* in ticks in the endemic regions, east of the Vistula River was similar (37.65 %; 451/1198) to prevalence in the non-endemic regions located between the Vistula River and the western border of the country (49.68 %; 689/1387) (NS).

**Fig. 2:**
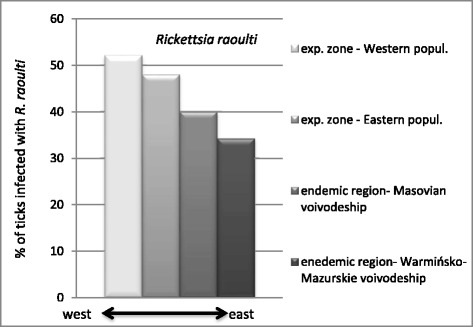
Prevalence of *R. raoulti* in *D. reticulatus* from the expansion zones (western Poland and west of the Vistula River) and from endemic regions (central and north-eastern Poland)

Interestingly, there was a marked increasing trend in the prevalence along an east–west geographic axis (Fig. 2). The highest prevalence was found, as mentioned above, in western Poland (western expansion zone) (52.03 %) and this declined eastwards. Prevalence was 47.92 % (381/795) in the expansion zone west of the Vistula River; 39.92 % (289/724) in the endemic region of the Masovian Voivodeship and 34.18 % (162/474) in the endemic region of the Warmińsko-Mazurskie Voivodeship in NE Poland. The differences in *Rickettsia* prevalence between the four geographical regions were significant (*χ*^*2*^ = 44.21, *df* = 3, *p <* 0.001) (Fig. 2).

As with *B. canis*, prevalence of *R. raoulti* differed significantly in a north-to-south direction in both expansion zones (in western Poland and west of the Vistula River) (*χ*^*2*^ = 17.44, *df* = 2, *p <* 0.001) but in western Poland the trend was reversed. The highest prevalence was found in northern districts of the western expansion zone - 70.21 % (33/47) with lower values for central and southern areas: 49.07 % (212/432) and 55.75 % (63/113), respectively. In the expansion zone in central Poland (west of the Vistula River), prevalence of *R. raoulti* in central (236/424 = 55.66 %) and southern (108/223 = 48.43 %) areas was about twice as high as in the northern districts (37/148 = 25 %).

### Comparison of parasite range between different tick populations

The number of pathogen species vectored by *D. reticulatus* was lower in the Western tick population (only *R. raoulti* was found) in comparison to the Eastern population (*B. canis, B. microti, B. afzelii, R. raoulti)*. Additionally, TBEV was detected in the Eastern tick population, although we made no attempt to identify this pathogen in ticks from the Western population. The range of vectored pathogens was similar in endemic regions east of the Vistula River (Masovian and Warmińsko-Mazurskie voivodeships) and in the adjacent central expansion zone (west of Vistula River).

Interestingly, there were marked differences in the prevalence of pathogens between the different sampling sites in the recently established expansion zones, especially regarding *B. canis* (0.68-14.8 %) in central and *R. raoulti* (25–70.2 %) in both western and central expansion zones.

## Discussion

The present study focused on pathogens vectored by *D. reticulatus* ticks in endemic regions and zones of expansion in Poland. Almost every second tick was infected by TBP’s (46.3 %), yet spatial diversity in prevalence of TBP’s was noted between ticks collected from the two isolated tick populations and among sampling sites in the expansion zones. It is highly likely that those two tick populations are of different origin. The tick population in eastern Poland is well known as a constituent of the Eastern macro region of *D. reticulatus* [12, 21]. New foci of this tick in the expansion zone in central Poland are a likely extension of this population and continuous with it. On the other hand, it is likely that ticks from western Poland originated from the adjacent population settled in eastern Germany [56, 57]. Because of the likely different origin of these tick populations, some differences in TBP’s prevalence as well as pathogen genetic diversity might be expected.

We found that differences in the prevalence of *B. canis* were the most obvious. All positive ticks were collected in eastern and central Poland (Eastern tick population) while all ticks from the West of the country were negative for this pathogen. The prevalence of *B. canis* in our study (4.18 %) as detected in questing adult ticks, is one of the highest on record. In general the prevalence of *B. canis* is higher in the Eastern macro region in comparison with the Western European one. In neighboring countries east of Poland prevalence of *B. canis* has been reported to vary from 0 % to 3.6 %. In a recent study conducted by Karbowiak et al. [58] 6 out of 205 (3.14 %) ticks collected in the Chernobyl exclusion zone in the Ukraine were positive, while in relatively close locations in southern Belarus, Reye et al. [32] did not detect any positive ticks among 142 questing adults and 124 ticks collected from animals. In ticks collected in Western Siberia in Russia *B. canis* was found in 3.6 % of *D. reticulatus* [55]. In the West European macro region the prevalence of *B. canis* has been reported to vary in the range 0–1.64 %. Reports from France (endemic region for canine babesiosis [1, 59, 60] are scarce but in a study conducted by Bonnet et al. [31] no *B. canis* DNA was found in 74 ticks collected in eastern France, while in western Germany prevalence was estimated at 2.5 % [61]. This region, as well as territory in northern Spain, Belgium and Netherlands has been inhabited by *D. reticulatus* in the last decades [62]. In these countries *Babesia canis* has been identified in 1 % [63], 0 % [64] and 1.64 % [65] of marsh ticks, respectively. Exceptionally high prevalence of *B. canis* infection in questing *D. reticulatus* ticks (83 %, 19/23) was reported by Schaarschmidt et al. [66] in the Swiss Midlands, another ‘expansion zone’ of marsh ticks. In this last study, the presence of *B. canis* was confirmed in 9 samples (39.1 %) by sequencing the 18S rRNA gene. In this region the first outbreaks of canine babesiosis were described at the beginning of the XXI^th^ century. The authors attributed such a high prevalence to the probable formation of microfoci of ticks. Such a focus, with an unexpectedly high prevalence (14.8 %), was also detected in our study among ticks collected in southern areas of the expansion zone in central Poland. The reasons for this phenomenon are not clear and need further investigation.

In our study, differences in the prevalence of *B. canis* in ticks were associated with the time of settlement of tick populations in particular areas. The differences in prevalence of *B. canis* in endemic regions and in the expansion zone that we recorded, correspond well with the results of a recent study conducted by Kubelová et al. [67] in Slovakia, where overall prevalence of *B. canis* was 3.2 %, and where prevalence of *B. canis* in *D. reticulatus* varied significantly between east (14.7 %), southwest (2.3 %) and west regions of the country (0 %). The authors hypothesized that this geo-spatial shift in prevalence of *B. canis* was a consequence of the spread of *D. reticulatus* from the south-east in a north-western direction. Svehlová et al. [68] confirmed a similar prevalence of *B. canis* in this tick species from southwestern Slovakia (1.8 %). This pattern raises questions about the possible mechanisms of emergence and settlement of canine babesiosis in non-endemic territories. One possibility is that uninfected ticks spread and survive in the newly colonized areas more effectively than carriers of *B. canis*. In order to fully understand the pattern of emergence of canine babesiosis, complex investigations are required of the interactions between the protozoan parasites, their vectors and the wild animals that may serve as reservoir hosts.

Differences in prevalence of *R. raoulti* between the two Polish tick populations were far less pronounced (52 versus 42 %). Although the two expansion zones are separated by a 260–300 km wide interval, the infection rates in zones were very much alike (52 versus 48 %). However, the pattern of north-to-south differences in prevalence of *R. raoulti* was reversed in these two zones. Also a difference in the prevalence of *R. raoulti* was evident in a west-to-east direction, with the highest infection rate in the Western population (52 %). This is consistent with studies conducted in area of Germany, west of Poland, where prevalence of this species is higher than in eastern and southeastern European countries. In southern Germany about 30 % of *D. reticulatus* have been found to be positive [69, 70], but increasing to 43 % and 67 % in the west (Saarland) and the east of the country (Saxony), respectively [70]. In Slovakia 26 % of ticks collected from vegetation were positive [71] and in Belarus 22 % [32]. The lowest prevalence (5 %) has been reported in the United Kingdom – an isolated island [36]. The range of prevalence of *R. raoulti* found in our study reflects well the results obtained by other authors in Poland. In the Lubelskie Voivodeship in eastern Poland 53 % (280/528) of *D. reticulates* were positive [72]. In north-eastern Poland Stańczak [73] found 41 % (116/285) of *D. reticulatus* ticks to be infected and Chmielewski et al. [74] detected *R. raoulti* in 57 % (34/60) ticks from Białowieża Primeval Forest National Park.

Differences in the prevalence of *B. canis* and *R. raoulti* can be explained by their dissimilar relationships with the vector. *Babesia* spp. have a complex life cycle involving ticks and vertebrate hosts. This protozoa undergoes sexual development in ticks and only gametocytes can survive in the midgut of the tick during the blood meal. Despite adaptive mechanisms, *Babesia* spp. affect the fitness of ticks [75]. In contrast, *Rickettsia* spp. are claimed to be possible endosymbionts of ticks with a commensal or even mutualistic association with their hosts [76, 77]. However, the ability of highly pathogenic *Rickettsia rickettsii* to reduce fitness and fertility of *D. andersoni* was also documented [78]. *D. reticulatus* ticks are competent reservoirs and vectors of *R. raoulti* although to-date no vertebrate hosts have been recognized as reservoir. The high prevalence of *R. raoulti* may be facilitated through transovarial and transstadial transmission which has been clearly demonstrated [79]. Given that the genetic diversity of these pathogens is known to be limited, confirmation of the primary origin of Polish Eastern and Western tick populations needs further investigation, and genotyping individuals from both regions of the *D. reticulatus* range, Western European or Eastern may help to resolve the issue.

Two species of *Babesia* were found in the current study: *B. canis* and *B. microti. B. microti* was detected only in one male collected in Warmińsko-Mazurskie Voivodeship, and this is the first report of this species in *D. reticulatus* from north-eastern Poland. Interestingly, Wójcik-Fatla et al. [80] found *B. microti* to be dominant over *B. canis* (2.1 % versus 0.7 %) in *D. reticulatus* ticks collected in the Lubelskie Voivodeship (eastern Poland). Some of the sequences detected were homologous to a genetic variant of the *B. microti* Munich strain, as in our study. High prevalence of this genotype (30-36 %) was observed also by Siński et al. [81] in *Microtus* spp. captured in the same region (Mazury Lake District, Warmińsko-Mazurskie Voivodeship). *Microtus* spp. voles are the main hosts for instars of *D. reticulatus* [82–84], and therefore trace amounts of *B. microti* DNA may be detected in this tick species. Interestingly, *B. canis* has low genetic variability in 18S rDNA, given that the same genotype was found in Croatia, Italy, Poland and southeastern Siberia. Elsewhere, *D. reticulatus* have been found to be infected also with other *Babesia* species [63, 65], however their role in the transmission of these pathogens to susceptible hosts needs further investigation.

Bacteria of the genus *Rickettsia* were the most prevalent pathogens found in *D. reticulatus* in all sampled regions with an overall prevalence 44.10 %. All 127 sequences derived from ticks collected in both expansion zones and endemic regions in Masovian and Warmińsko-Mazurskie Voivodeships were identical and homologous to *R. raoulti.* This species was detected for the first time by Rydkina et al. [85] in Russia and it was described by Mediannikov et al. [86] as a novel species. Subsequently this intracellular bacterium has been classified in the spotted fever group (SFG) of *Rickettsia*. Together with *R. slovaca* and *R. sibirica*, *R. raoulti* is thought to be responsible for tick-borne lymphadenopathy (TIBOLA), a syndrome that is manifested mainly by eschar around the tick attachment sites and enlargement of the nearest lymph nodes. Cases of TIBOLA caused by *R. raoulti* have been reported from Spain [87], France [86, 88], Germany [89], Hungary [29] and Poland [90]. Földvári et al. [29] connected 6 cases of TIBOLA in humans to the bites of *D. reticulatus*, having amplified *R. raoulti* DNA from 5 females and 1 male *D. reticulatus* collected from patients with this syndrome.

Evidence for the pathogenicity of *R. raoulti* for animals is so far lacking. Moreover, only 2.8 % of dogs from Germany, examined with the micro-IFA test, have been found to be seropositive for *R. raoulti* [91] in contrast to *R. helvetica* (66 %). Other tick species have been found also to harbour *R. raoulti*, e.g., *Rhipicephalus pumilio*, *D. nutalli* [85], *D. marginatus* [92], *I. ricinus* [74]. However, *D. reticulatus* appears to be the main vector involved in transmission of the bacteria in Poland and other countries where it occurs, as reflected in the high prevalence of *R. raoulti* in our and other studies.

The potential role of *D. reticulatus* in the maintenance and circulation of TBEV and a link with cattle as reservoir hosts has been demonstrated in recent studies [38, 39], including ours. The prevalence of TBEV estimated in our study (7.6 %) is consistent with the results obtained by Wójcik-Fatla et al. [38] in tick samples from the Lubelskie Voivodeship (10.8 %). In a study conducted by Biernat et al. [39] the prevalence of TBEV varied across northeastern and central Poland (0.99-12.5 %) and it is known to vary also between susceptible tick species. For example, prevalence of TBEV in *D. reticulatus* may be up to 10 times higher than in *I. ricinus* (7-11 % versus 0–1.2 %; [38, 93]). Cattle have been shown to be competent hosts for *D. reticulatus* and the dominance of this tick over *I. ricinus* on bovine hosts in endemic regions has been reported recently [28]. Grazing cattle may play a dual role; they serve as an easily available source of blood meal compared to wild animals thus supporting the expansion of *D. reticulatus* and act as a reservoir for the tick-borne encephalitis virus (TBEV). Transmission of TBEV to cattle may be followed by transfer of this virus to humans via non-pasteurized milk or other dairy products from infected animals (mainly goats, sheep and cows) [94]. Milk-borne TBE outbreaks or single cases have been reported from Eastern Europe, Austria and Germany [95].

Low prevalence of *B. burgdorferi* s.l. in *D. reticulatus* has been reported in many earlier studies including our own (0.09 %): 0 % in Germany [35], United Kingdom [36] and Serbia [33], 0.6 % in the Lubelskie Voivodeship in Poland [96], 1.5 % in France [31], 2.7 % in Belarus [32] and *Borrelia afzeli* has been the most often detected genotype of the *B. burgdorferi s.l.* complex, as also in our study. However, in contrast to these low overall prevalence rates, this genotype has been reported with a much higher prevalence in engorged nymphs of *D. reticulatus* removed from *Microtus* spp., which are known to be reservoir host for *B. afzeli* (Bajer unpublished). An explanation for these contrasting prevalence rates can be found in reports that indicate a rapid drop in the infection rate with *Borrelia* spp. in *D. reticulatus* shortly after feeding [97]. Extracts from the midguts of *D. reticulatus* have been shown to inhibit the growth of *Borrelia* spirochetes in vitro [98] and an active immune response against *Borrelia* bacteria, leading to lysis of the spirochaetes, has been demonstrated in *D. variabilis* [99]. Based on these findings, it is likely that the fall in the prevalence of *B. burgdorferi* s.l. in adult *D. reticulatus* is a consequence of the inactivation of bacteria by the immune system of the ticks. Therefore *D. reticulatus* are not competent vectors for the *Borrelia burgdorferi* s.l. complex [100] and it is unlikely that this tick is involved in transmission and epidemiology of Lyme boreliosis.

## Conclusions

Prevalence of TBP’s transmitted by *D. reticulatus* in Poland depends on the region of study. The region with the highest probability of transmission of *B. canis*, in both the endemic region and expansion zone, is the Masovian Voivodeship. Due to the high prevalence of *R. raoulti*, *B. canis* and TBEV, the endemic regions of the Masovian Voivodeship are at the greatest risk of diseases caused by TBP’s of medical and veterinary importance. *R. raoulti* is the most prevalent pathogen harbored by *D. reticulatus* and may be the main cause of TIBOLA in Poland.
